# Metro Maps of Plant Disease Dynamics—Automated Mining of Differences Using Hyperspectral Images

**DOI:** 10.1371/journal.pone.0116902

**Published:** 2015-01-26

**Authors:** Mirwaes Wahabzada, Anne-Katrin Mahlein, Christian Bauckhage, Ulrike Steiner, Erich-Christian Oerke, Kristian Kersting

**Affiliations:** 1 INRES-Phytomedicine, University of Bonn, Bonn, Germany; 2 Fraunhofer IAIS, Sankt Augustin, Germany; 3 B-IT, University of Bonn, Bonn, Germany; 4 CS Department, Technical University of Dortmund, Dortmund, Germany; College of Agricultural Sciences, UNITED STATES

## Abstract

Understanding the response dynamics of plants to biotic stress is essential to improve management practices and breeding strategies of crops and thus to proceed towards a more sustainable agriculture in the coming decades. In this context, hyperspectral imaging offers a particularly promising approach since it provides non-destructive measurements of plants correlated with internal structure and biochemical compounds. In this paper, we present a cascade of data mining techniques for fast and reliable data-driven sketching of complex hyperspectral dynamics in plant science and plant phenotyping. To achieve this, we build on top of a recent linear time matrix factorization technique, called Simplex Volume Maximization, in order to automatically discover archetypal hyperspectral signatures that are characteristic for particular diseases. The methods were applied on a data set of barley leaves (*Hordeum vulgare*) diseased with foliar plant pathogens *Pyrenophora teres*, *Puccinia hordei* and *Blumeria graminis hordei*. Towards more intuitive visualizations of plant disease dynamics, we use the archetypal signatures to create structured summaries that are inspired by metro maps, i.e. schematic diagrams of public transport networks. Metro maps of plant disease dynamics produced on several real-world data sets conform to plant physiological knowledge and explicitly illustrate the interaction between diseases and plants. Most importantly, they provide an abstract and interpretable view on plant disease progression.

## Introduction

Plant diseases are responsible for the loss of at least 10% of the global food production thus exacerbating the problem of food shortages in a world where at least 800 million people suffer from malnutrition [1, 2]. Plant diseases are therefore a key challenge in crop production and require close attention. However, understanding and modeling the response behavior of diseased plants is itself a challenging task. Pathogenesis results from complex interactions between the genotype of the plant, the pathogen, and the environment leading to disease symptoms. While genetic and biochemical approaches towards understanding stress reactions are constantly improving, *phenomic approaches* which measure the structural and functional status of plants have recently been found to overcome the limited predictive capabilities of current methods [3] and form an instance of the current trend of *“modeling the life on earth”*[4].

A particularly promising approach towards bridging the gap between current “omic”-technologies, such as transcriptomics, genomics, metabolomics and phenomics [5–7] consists in non-invasive, hyperspectral imaging (HSI). This kind of *sensor-based phenotyping* has already been proven successful in monitoring physiological traits and plant genotype specific responses to biotic and abiotic stresses [7,8]. Currently, different automated phenotyping platforms are available which comprise automated handling and organization of experimental plants, innovative technologies from robotics, and new sensors and imaging technologies and therefore allow for a wide range of applications, ranging from lab research to screening systems on the field scale [6,9]. However, state-of-the-art sensor techniques and increasing amounts of sensor data lead to new challenges in plant science, because data needs to be interpreted and related to plant traits. Ultimately, quantitative and qualitative plant traits have to be determine automatically in order to achieve high-throughput phenotyping. This requires efficient and reliable analysis techniques which avoid costly and time consuming manual interventions.

Information as to plant health and plant physiology can be inferred from nearly any part of the electromagnetic spectrum. For instance, the visible region (VIS) from 400 to 700 nm is influenced by the pigment content, the near infrared (NIR) reflects structural characteristics of plants, and the shortwave infrared (SWIR) mainly reflects leaf chemical components and water content [10]. Plant diseases, in turn, influence these biophysical and biochemical properties during pathogenesis as they cause variation of pigments and leaf structure due to chlorosis, necrosis and fungal structures on the leaf surface. These changes will result in specific spectral signatures which vary dynamically during disease development in plants and crop stands. Therefore, phenotyping processes may benefit from hyperspectral analysis and corresponding models of disease dynamics.

**Figure 1:**
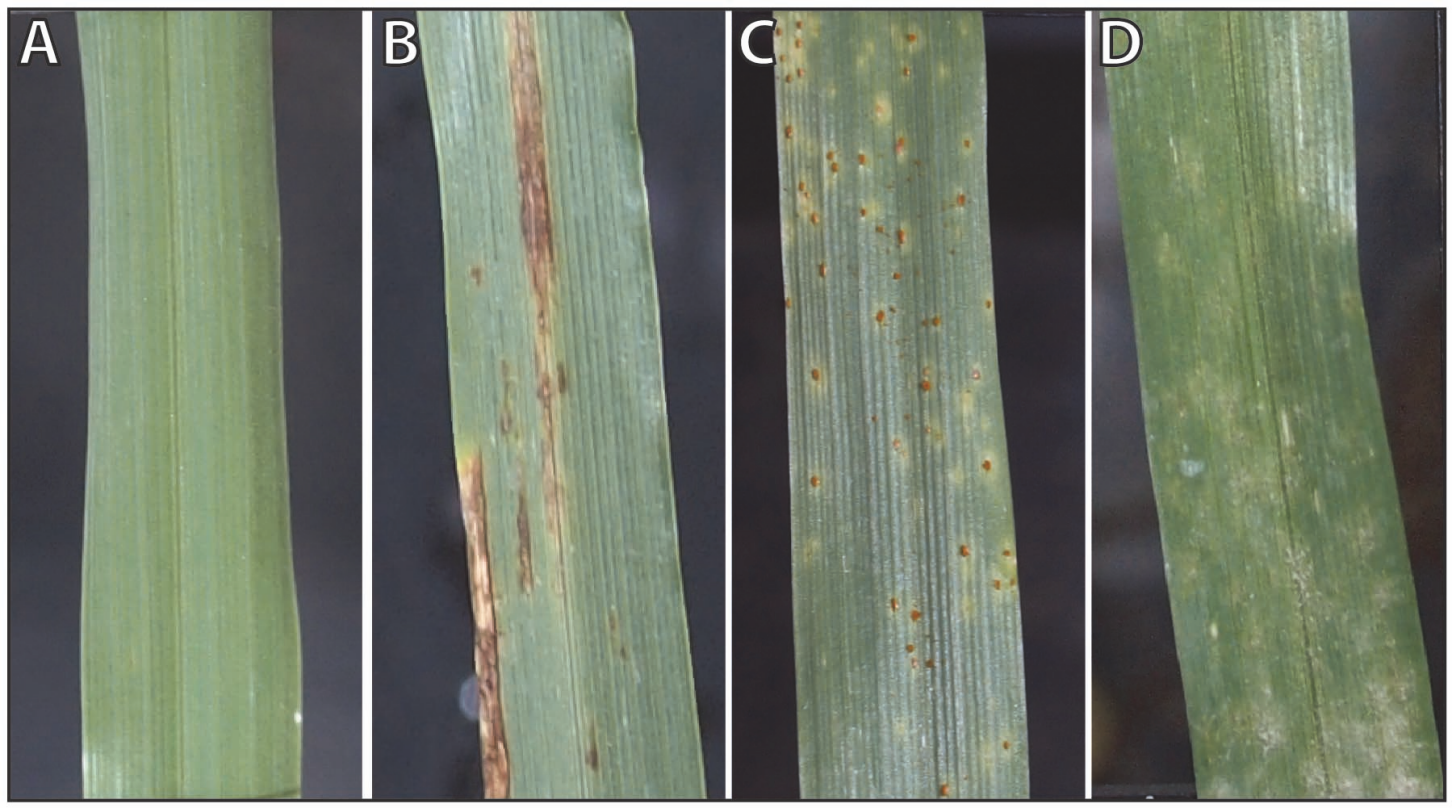
Characteristic phenotypes of healthy and diseased barley leaves. (**A**) Healthy barley leaf, (**B**) net blotch caused by *Pyrenophora teres*, (**C**) brown rust caused by *Puccinia hordei*, and (**C**) powdery mildew caused by *Blumeria graminis hordei*.

Barley (*Hordeum vulgare*), an important crop for food and feed production, may suffer from several economically relevant leaf diseases during the vegetation period, resulting in a significant loss in yield and grain quality [11]. Net blotch, brown rust and powdery mildew are destructive pathogens in barley production. A timely detection of primary disease foci in the field is therefore vital for effective plant protection strategies. As the examples in Fig. 1 show, each of these diseases exhibits distinct symptoms and affects the host plant metabolism and function in a specific way [12]. This results in characteristic spectral reflectance signatures. In order for hyperspectral sensors to support an automated detection of plant diseases, characteristic spectral dynamics during symptom development need to be known and information available from hyperspectral imaging should be visualized in an intuitive way.

Yet, any automated analysis of biological processes based on hyperspectral imaging imposes challenges with respect to scalability and interpretability. For instance, scientists working on plant phenotyping frequently encounter the problem of having to deal with massive, high-dimensional, and temporally varying observations contained in large collections of hyperspectral data cubes (as used in the present study). Moreover, put in colloquial terms, today’s “large” is tomorrow’s “medium” and next week’s “small”. Finally, in addition to multispectral and hyperspectral sensors first ultraspectral sensors are coming into operation. Hence, in addition to the problem of optimization of technical solutions for automated phenotyping, research has to address the problem of interpretation and handling of phenomic data. Motivated by these observations, we approached the challenge of how to efficiently analyze huge amounts of hyperspectral data in phenomics. In particular, we investigated the question “Can machines automatically provide interpretable summaries of plant diseases progressions at massive scale?” This issue of “interpretability” is pivotal in our work since plant phenotyping is necessarily an interdisciplinary endeavor where scientists with complementary skills work together. Intuitively, interpretable summaries, which we call *sketches*, can be defined as a description of the hyperspectral dynamics of a plant disease, consisting of one (single sketch) or several paths (metro maps) which describe the development or highlighting interesting moments of diseases. The sketches are drawn at different points in time during disease development and are based on so-called “disease archetypal hyperspectral signatures”. These signatures characterize extremal measurements for a particular disease and therefore differ from the signatures of other diseases or healthy plants. Moreover, they can be discovered automatically at massive scale while taking into account any given information such as hyperspectral images or manually assigned labels.

The above challenges make it difficult to use off-the-shelf statistical techniques such as Principal Component Analysis, Hidden Markov Models, Support Vector Machines, or Gaussian Processes for analyzing sequences of hyperspectral images: they often do not provide easy-to-interpret features or models and —unless one resorts to approximations that entail information loss— they typically do not scale well to large amounts of data. In this paper, we therefore build on recent, fast data-driven approaches for mining *abiotic* stress signals [13–16] that meet the challenges of interpretability and scalability.

The main objectives of the presented study are (i) to automatically extract disease specific spectral signatures over time (archetypal signatures), (ii) to link the spectral dynamics to biological processes during pathogenesis, and (iii) to visualize disease progression by sketching the hyperspectral dynamics in an easily interpretable and intuitive way using metro maps. In order to achieve these goals, we adapt machine learning and data mining methods for a fast and accurate analysis and apply a recent linear time, easy-to-parallelize data driven approach to obtain suitable representations of the data.

## Results and Discussion

**Figure 2:**
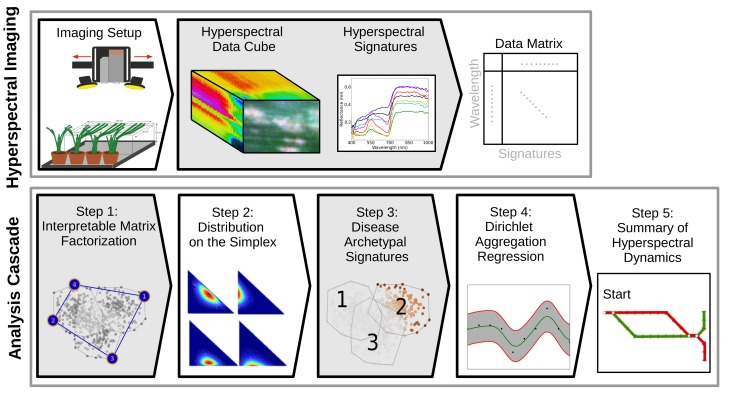
Mapping disease progression in plants at massive scale. Consecutive steps from hyperspectral imaging data of healthy and diseased barley leaves to interpretable summaries by metro maps in five consecutive steps. (Best viewed in color)

The procedure for creating interpretable summaries can be briefly decomposed in five consecutive steps that are summarized in Fig. 2 (bottom). We start by determining a new representation using interpretable matrix factorization (Step 1). That is to say, based on a data set of hyperspectral images of healthy and diseased barley leaves that were recorded during disease development, representative data are automatically determined by Simplex Volume Maximization across all images. This provides us with global characteristics of healthy and diseased leaves. Then, each hyperspectral signature is expressed as a combination of these representatives, leading to a parametric distribution (Step 2) for each hyperspectral image. These representations capture local dependencies. Based on the estimated parameter distributions, we automatically discover disease archetypal signatures using Bayes factors (Step 3) and capture their dynamics using Dirichlet Aggregation Regression (Step 4). Finally, this information is used for visualizing plant disease dynamics in form of metro maps (Step 5).

The intention behind this approach is to investigate our main question (MQ): Do automatically produced sketches of hyperspectral dynamics conform to plant physiological knowledge and produce a characteristic and plausible picture of disease progress and symptom appearance? Additionally, we investigate the supporting question (SQ): Do the selected disease archetypal signatures distinguish between plants with different diseases better then signatures that neglect available disease label information?

### Evaluation of Disease Archetypal Signatures

**Figure 3:**
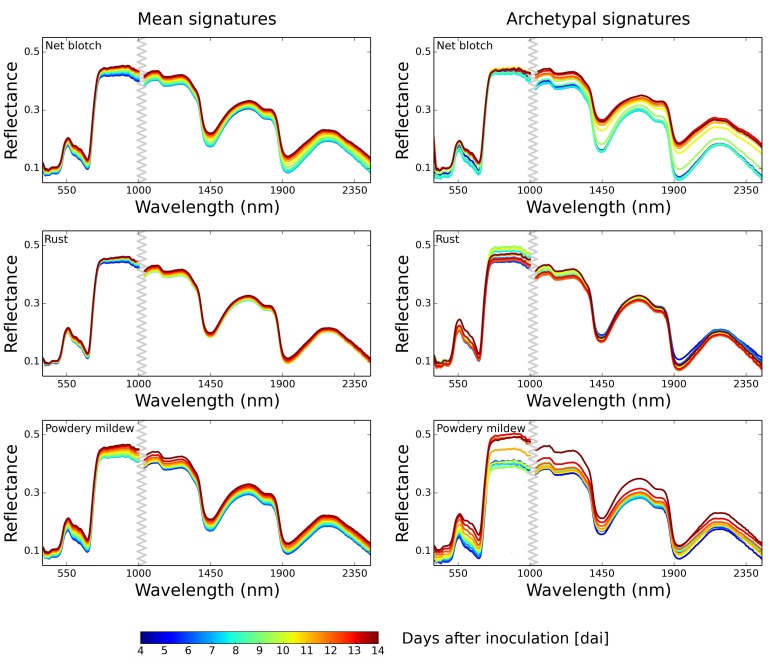
Interpolated mean signatures and archetypal signatures for visible-near infrared (VNIR) and shortwave infrared (SWIR) wavelengths (measured 4–14 dai). In the left column mean signatures of diseased barley plants before selecting disease archetypal signatures and in the right column mean archetypal signatures for *η* = 1 are illustrated. Archetypal signatures allow a better differentiation between different developing stages of the diseases. Moreover, they are in accordance to visually and manually extracted reflectance signatures during disease development. (Best viewed in color)

**Figure 4:**
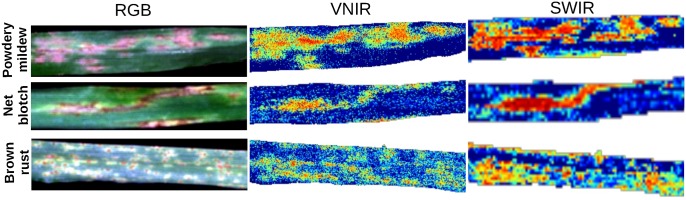
Disease archetypal selections. An example image showing diseased barley plants (RGB, first column) with powdery mildew (first row), net blotch (second row) and rust (third row) 14 dai. False color images present automatically determined diseased plant pixels based on disease archetypal signatures for VNIR and SWIR data (middle and right columns). The yellower/redder the color, the greather the difference of the pixel to a healthy plant. (Best viewed in color)

To answer questions (MQ) and (SQ) we compared hyperspectral signatures before and after a selection of disease archetypal signatures. Automatically determined Dirichlet mean signatures per day are shown in Fig. 3 where the Dirichlet mean signatures in the left column were obtained prior to a selection of disease archetypal signatures, and the mean signatures in the right column were determined after selecting disease archetypal signatures. Disease archetypal signatures only consider pixels which are relevant and characteristic for a disease (Fig. 4), healthy pixels are neglected. Thus they constitute an accurate measure for a differentiation among diseases (Fig. 3, right column). Characteristic changes in the spectral reflectance are more distinct compared to regular mean signatures extracted from entire leaves (Fig. 3, left column). These archetypal signatures are additionally useful for distinguishing among different time points during pathogenesis which is discussed below. That is, each pathogen caused a characteristic disease progress and symptom appearance on barley plants and the following cold be deduced from archetypal signatures:

Net blotch, caused by the necrotrophic pathogen *P. teres* appeared 4 dai (days after inoculation). Small brown and necrotic spots appeared at the infection site 24 h after penetration [17]. The early symptoms increased reflectance in the VIS from 550 to 700 nm, slight changes in the NIR were observable (Fig. 3, first row). These lesions grew along leaf veins forming a net-like pattern. Over time, the surrounding tissue became chlorotic and water-soaked which may be caused by diffusible toxins or effectors secreted from fungal hyphae [17]. Physiological changes during pathogenesis were well reflected in automatically assessed disease archetypal signatures. Increase in reflectance in the visible range resulted from local loss of chlorophyll when necrosis appeared; similar effects are reported by [12] for symptom development of *Cercospora beticola* in sugar beet. Symptom development is observed to proceed steady going over time, changes in reflectance were more gradually than escalating, correlating to the stage of the diseases, respectively. In later stages of pathogenesis, barley tissue became necrotic; the loss of water in symptomatic areas influenced the spectral reflectance in the SWIR-region (Fig. 3, first row) [18].

Within the first days after inoculation of *P. hordei*, small chlorotic rust spots appeared on the leaf surface, resulting in an increase of reflectance in the range 550–700 nm, as shown in Fig. 3, second row. This reflectance change confirmed results from Teng and Close [19] who measured reflectance of barley leaves differing in rust severities. The chlorotic spots proliferated until 6 dai. Between 6 and 8 dai, accumulation of orange brown uredino spores under the epidermis became visible at infection sites. Explicit changes in spectral signatures were observed 12 dai, when the epidermal layer ruptured, and the rust spores became visible on the leaf surface, causing a reflectance shift peaking at 520 nm. Teng et al. [19] stated that the reflectance of rust infected leaves is a function of the erupted uredina and sub-epidermal fungal growth. The reflectance increase around 600 nm resulted from the high amount of orange-brown rust spores on the leaf [20, 21]. However the impact of *P. hordei* on leaf structure and on the water content of the tissue was comparatively low. This was clearly apparent in the NIR and SWIR of the archetypal signatures.

Powdery mildew causes white, fluffy pustules distributed on the upper and lower leaf side. The first pustules caused an overall increase of reflectance in the VIS. Similar effects on leaf reflectance of barley were observed in barley fields diseased with powdery mildew [22, 23]. This parallel shift of the reflectance was clearly described by archetypal signatures (Fig. 3, third row). The size and number of these symptoms increased within 10 dai, resulting in changes of NIR reflectance. As a biotrophic pathogen, *B. graminis hordei* is establishing a long-term feeding relationship with living host plant cells [24]. After ten days of powdery mildew infection, the tissue next to the colonies became chlorotic and finally necrotic. This conspicuous impact on leaf structure and leaf phenology was reflected in archetypal signatures, where the strongest differences occured in the period 10 to 14 dai [22]. At this time point, the SWIR-reflectance was significantly higher due to a successive loss of water at and around lesions.

Additionally, we computed the accuracy of correctly classified signatures using training examples from all as well as only from disease archetypal selections using the nearest convex hull classification approach [25]. The accuracy results for the test set with all data showed that taking training examples from disease archetypal signatures can significantly outperform those from all data, indicating a better differentiation between diseases. We note, classification was not the goal in this work. Here we only investigated whether disease archetypal signatures allow for differentiating between different diseases. For classifier training, we only considered labels for whole images only and did not consider whether individual signatures/pixels represented healthy or diseased parts of a leaf. Nevertheless, the classification of diseased plants using sensor data such as hyperspectral images is of considerable practical interest. We leave this question for the future work.

### Mapping Disease Progression

Hyperspectral reflectances differed between net botch, rust, and powdery mildew depending on the developing stages of the diseases. This was clearly visible in disease archetypal signatures. Regarding question (MQ), we further asked at which point in time during pathogenesis relevant dynamics and changes in reflectance become apparent? We therefore provide computed summaries by sketching hyperspectral dynamics of diseased plants which we will discuss in detail in the next subsections.

#### Disease Progression via Single Sketches

**Figure 5:**
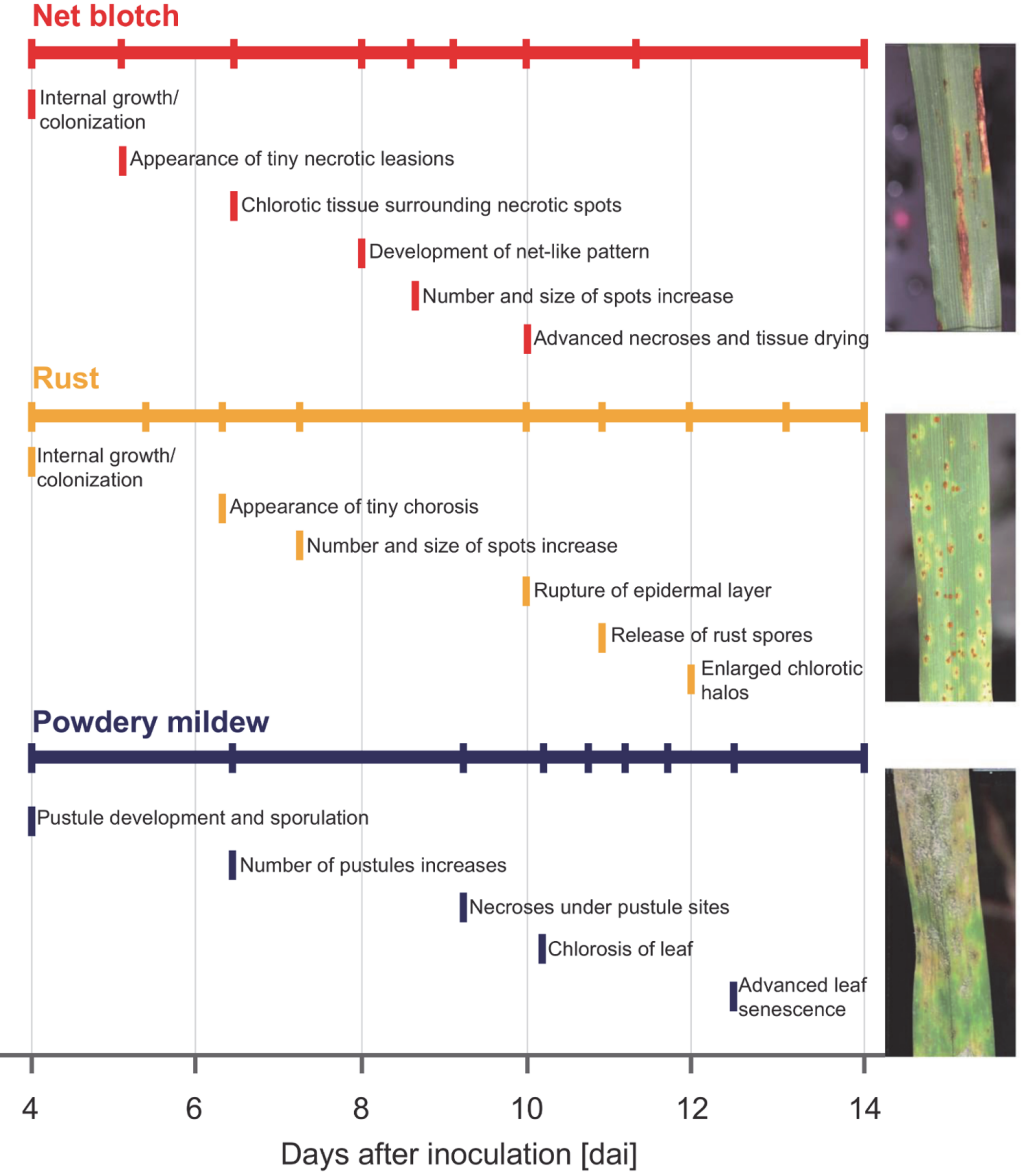
Single sketches of hyperspectral dynamics of plant diseases for visible-near infrared (VNIR) wavelengths. Each sketch consists of parts encoding major states during pathogenesis of the plant disease with similar weights. Thus, the shorter a part, the higher the impact of the corresponding period. (Best viewed in color)

for the VNIR ranges, single sketches of the barley diseases based on mean reflectance archetypes are shown in Fig. 5 (for the sake of clarity, the figure omits results for SWIR ranges, which are provided in supporting information). Each sketch consists of parts (line segments) which encode major stages of the dynamics using similar weights. Thus, the shorter a part, the higher the impact of the corresponding period.

Major states of the dynamics appeared to differ between diseases: Net blotch diseased leaves exhibited a steady development in the first 7 days. From 7 dai to 12 dai significant reflectance changes appeared in the VNIR and SWIR ranges which corresponds to biological processes during pathogenesis. First symptoms are chlorosis and small necroses 4 dai to 7 dai, reflected in changes in the VNIR. Severe structural and biochemical changes of the leaf tissue occur 7 dai to 12 dai, and a considerable water loss can be deduced from a period with high impact in the sketch of the SWIR data. The impact of rust on barley leaves was comparatively minor and more consistent over time. The biotrophic pathogen aims to feed from living host cells to produce new rust spores. Time points with crucial processes appear in the VNIR between 5 dai to 7 dai where first chloroses appeared and changed into small brownish rust pustules. Starting 10 dai, the rust spores ruptured the epidermis and massive amounts of spores were released from each pustule; this relevant step during rust pathogenesis was clearly marked in the sketches (Fig. 5). The dynamics of rust pathogenesis in the SWIR were minor as the effect depends on the biotrophic lifestyle of *P. hordei*. The characteristic powdery mildew pustules on the leaf surface constantly grew during the first 9 days of pathogenesis. Notable changes in pustule size could be recorded; however, the color of powdery mildew colonies stayed whitish and the surrounding barley tissues seemed to remain intact to the greatest possible extent. From 9 dai onwards, the color of the powdery mildew mycelium changed from whitish over gray to brownish. This process can be traced in the sketches of hyperspectral dynamics of powdery mildew (Fig. 5). Barley tissue became necrotic beginning 12 dai, and, in turn, SWIR reflectance increased noticeably. The sketches based on disease archetypal signatures highlight relevant steps during disease progression. This characteristic information is hidden when using mean signatures without archetypal selection.

#### Metro Maps of Disease Dynamics

**Figure 6:**
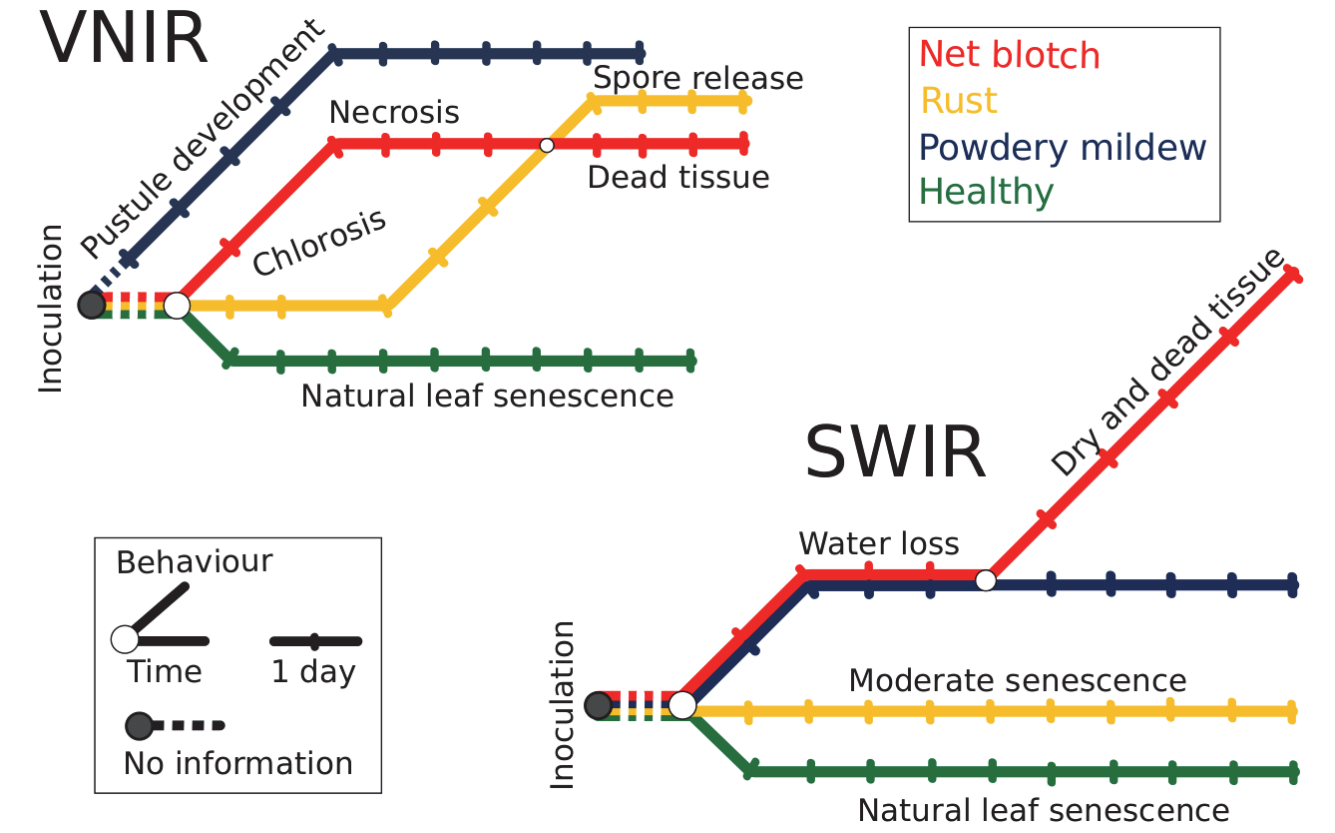
Collective disease progression via Metro Maps of hyperspectral dynamics of diseased plants for visible-near infrared (VNIR) (top) and short-wave infrared (SWIR) wavelengths (bottom). Each disease track from hyperspectral images exhibits a specific route in the metro map, the direction and the dynamic steps are in correspondence to biophysical and biochemical processes during disease development. The beginning of all routes is at the same time point/train station (day of inoculation, gray circle). (Best viewed in color)

So called metro maps provide an innovative and intuitive way to illustrate spectral dynamics and changes during pathogenesis (Fig. 6) as they can be seen as a concise structured set of salient information. Metro maps of disease development therefore provide a metric of the interaction between each pathogen and the host tissue as well as a simply structured summary of the spectral information during disease development. The connectivity of a map conveys the underlying biophysical and biochemical processes during disease development as well as similarities between the three diseases at different developmental stages.

Each disease track exhibits a specific route in the metro map. The start of all routes is set at the same time point/train station (day of inoculation, gray circle). From the first days after inoculation the appearance of powdery mildew on barley is different to that of other diseases; the relatively intact tissue is covered by white mycelium colonies producing an increasing amount of conidia with a dominant influence on the VNIR spectrum (Fig. 6, top). Net blotch and rust share the occurrence of chloroses in early stages of disease development. However, net blotch causes early necrotic tissue damage and followed a different path until 10 dai; here, the routes of rust and net blotch disease are interlaced in the VNIR, as the epidermis of rust diseased barley leaves is ruptured and thousands of brownish rust spores are released. The path of healthy barley plants has a different general direction than the other treatments in the VNIR and SWIR metro map, because only natural senescence processes influence the spectral course of healthy barley plants (Fig. 6). SWIR spectral reflectance is mainly influenced by water content. During rust pathogenesis only tiny chlorotic spots with rust uredina appear on the tissue; necrosis and loss of water occurred only at later stages and were not present in our experiments. Thus, the deviation of the rust track from the one of healthy plants is minor (Fig. 6, bottom). A major impact on SWIR reflectance by powdery mildew and net blotch was observed and described by archetypal signatures. Metro maps are an easily interpreted graphical image of this effect. The course of powdery mildew and net blotch is summarized in a similar manner until day 9, afterwards the destructive characteristic of net blotch affects SWIR reflectance to a greater extent than powdery mildew which causes rather local necrosis and tissue damage.

#### Discussion of Methodology

The present paper provides a novel and efficient data analysis cascade for plant phenotyping data recorded from plants diseased with different pathogens. The developed framework aims at an automatic information extraction from hyperspectral images and at an intuitive visualization of results; a particular focus is on potential practical impact and the potential for implementation in automated phenotyping platforms.

The benefits of the presented sketching approaches, summarized in Fig. 2, are manifold: Metro maps are natural tools for passengers to navigate in large urban areas [26]. Because people easily read and understand such maps, the metro map metaphor currently emerges as an easy-to-understand technique for visualization of abstract interconnected “trains of thoughts” [27] and as a tool to automatically construct structured summaries of information in scientific texts or news articles [28]. An additional favorable property is that metro maps illustrate relations between case lines which, in our study, are the dynamics of healthy and diseased barley leaves. Single sketches as well as metro maps are expressed in terms of disease archetypal signatures which carry meaningful information for plant biologists. They conform to plant physiological knowledge, explicitly illustrate the interaction between plants and pathogens, and provide an interpretable summary of disease progressions.

Disease archetypal signatures can be discovered at massive scale by using a recent linear time matrix factorization approaches, which re-parameterizes each hyperspectral image of a disease (a data matrix) in terms of a distribution on the simplex spanned by a few extreme signatures (a small number of pixels in the hyperspectral images). This facilitates high-throughput phenotyping and avoids running the risk of loosing information when selecting typical signatures manually at some diseased spots only. Since extreme data points (approximately) form a simplex, there are natural candidates to describe their statistical distribution, namely simplex distributions such as the Dirichlet distribution. In contrast to other “data cloud” embedding methods, see e.g. [29], we therefore do not make any assumptions as to the true generating distribution of each input data matrix, which is good practice since these are typically unknown. In contrast to histogram based embedding approaches such as, for instance, proposed by Sakurai *et al.*[30], probabilistic inference can be performed to quantify the “impact” of extremes in a dispersion model or to determine the “distance” between the data matrices in an information theoretic manner, cf. Kersting *et al.*[14]. Generally, distributions pave the way to statistical data analysis methods such as regression techniques, similarity metrics, low-rank embeddings, and advanced visualization techniques.

To summarize, linking disease symptomatology with disease archetypal signatures and metro maps of the diseased plants clearly shows that questions (MQ) and (SQ) can be answered affirmatively.

## Materials and Methods

### Data Description

#### Plant Material and Pathogens

A data set of hyperspectral images recorded from barley plants formed the basis for further analysis in this research. Barley plants (*Hordeum vulgare* cv. Leibniz, KWS Lochow, Bergen-Wohlde, Germany) grown in a controlled greenhouse environment were used for hyperspectral measurements after reaching growth stage (GS) 32 [31]. For each pathogen treatment, 9 plants were inoculated with *Pyrenophora teres* (causing net blotch disease), *Puccinia hordei* (causing leaf rust of barley) and *Blumeria graminis hordei* (causing powdery mildew), respectively. A control group was kept non-inoculated. Conidia of *P. teres* were harvested from diseased barley leaves, sampled from fields during spring and incubated in a moist chamber for 24 h at room temperature. *Pyrenophora teres* was inoculated by spaying a spore suspension (1×10^4^ conidia *ml* ^−1^) onto leaves using a hand sprayer. Subsequently, plants were placed in transparent plastic boxes to realize 100% relative humidity (RH) at 23/20^∘^C for 24 h. Uredospores of *P. hordei* for inoculation were obtained of from diseased leaves and were stored at 4^∘^C. Suspension of *P. hordei* (4×10^4^ uredospores *ml* ^−1^) were sprayed onto leaves before placing the barley plants in plastic boxes and incubating them for 24 h at 23/20^∘^C and 100% RH. After 24 h the plants were removed from the plastic boxes. For inoculation of *B. graminis hordei*, conidia from sporulating colonies were distributed over barley leaves in a ventilator chamber. After incubation, all plants were kept in the greenhouse at 21/18^∘^C and 60% RH, whereas *B. graminis hordei* inoculated plants were kept in an extra chamber to avoid cross-contamination of the other treatments. Disease progress of the foliar pathogens *Pyrenophora teres*, *Puccinia hordei*, and *Blumeria graminis hordei* were observed for 2 weeks after inoculation. For each treatment 9 leaves (each belonging to a different plant, respectively) were assessed at each measuring day.

#### Hyperspectral Imaging and Data Representation

Hyperspectral images of barley leaves were recorded with two line scanning spectrographs 4, 6, 8, 10, 12 and 14 days after inoculation (dai). The ImSpector V10E (Spectral Imaging Ltd., Oulu, Finland) covers the VIS and the NIR ranges from 400 to 1000 nm, with a spectral resolution of up to 2.8 nm and a spatial resolution of 0.12 mm per pixel, resulting in 210 hyperspectral bands. A SWIR-camera (Spectral Imaging Ltd., Oulu, Finland) was used to record hyperspectral images in the SWIR ranges from 1000 to 2500 nm, with a spectral resolution of 5.8 nm and a spatial resolution of 0.4 mm per pixel, resulting in 226 bands. Constant illumination was provided by six ASD-Pro-Lamps (Analytical Spectral Devices Inc., Boulder, USA). The hyperspectral cameras and the illumination system were installed on a motorized line scanner (Spectral Imaging Ltd., Oulu, Finland) to obtain a second spatial dimension. The camera settings and the control of the motorized line scanner were adapted using the SpectralCube software (Spectral Imaging Ltd., Oulu, Finland). The hyperspectral images were recorded in a dark chamber in order to realize constant and reproducible illumination and measurement conditions. For a detailed description of the measuring setup, see Mahlein *et al.* [12]. Normalization and smoothing of raw hyperspectral images were performed using the software ENVI 4.6 + IDL 7.0 (EXELIS Visual Information Solutions, Boulder, USA). Reflectance was calculated relative to a white reference bar and to dark current measurement. Then the Savitzky-Golay filter [32] was applied to remove noise and to smooth the spectral information of the hyperspectral images. Furthermore the background of the images was masked applying band thresholds of R551 nm < 0.045, R667 nm > 0.085 and R798 nm < 0.8 in the VNIR, and of R1124 nm < 0.1 in the SWIR (R = reflectance at wavelengths indicated).

For subsequent analysis, each hyperspectral image was represented as dense Λ×*N* matrix, where *N* denotes the number of pixels and Λ the number of spectral bands (see Fig. 2 (top) for an abstract illustration). By stacking all data matrices recorded during pathogenesis, we obtained a data matrix with approximately 10 millions columns or about 2 billion matrix entries (encoding the reflected energy at different spectral bands) for the VNIR and about 200 million entries for the SWIR data set. Furthermore, each hyperspectral data set contains temporal information (day after inoculation) and one of the four labels *“healthy”*, *“rust”*, *“powdery mildew”* and *“net blotch”* which were used throughout the analysis in this work.

### From Hyperspectral Data to Interpretable Summary

This section covers the methodological part of this work and we describe the steps of the analysis cascade shown in Fig. 2. The starting point of our analysis are hyperspectral images represented as dense data matrices. In the following, we motivate the use of interpretable matrix factorization techniques [33] and discuss how they lead to parametric distributions. Given these parameters, we can automatically discover disease archetypal signatures using Bayes factors and capture their dynamics using Dirichlet Aggregation Regression [15]. Finally, we present further details on creating interpretable summaries. That is, steps (3) and (5) in Fig. 2 (bottom) and on how to devise the overall cascade that produces interpretable summaries that are the main algorithmic contributions of the present paper.

#### Step 1: Interpretable Matrix Factorization

In the first step of our framework, we employ interpretable matrix factorization at massive scale. Matrix factorization methods allow for embedding high dimensional data in lower dimensional spaces and can therefore mitigate effects due to noise, uncover latent relations, or facilitate further processing and ultimately help finding patterns in the data set distribution. More precisely, they factorize a matrix $X\in {\mathbb{R}}^{m\times n}$ into two matrices

$$X\approx \hat{X}=WH,$$ (1)

where the matrix of basis vectors $W\in {\mathbb{R}}^{m\times c}$ and the coefficient matrix $H\in {\mathbb{R}}^{c\times n}$ containing the low dimensional coordinates, are typically determined by minimizing a cost function, such as the squared Frobenius norm. A widely used technique for the factorization in Eq. (1) is Principal Component Analysis (PCA) [34] which is known to retain as much of the variation present in the data as possible. It is effective in terms of data compression, noise and redundancy reduction and typically projects the data points into a lower dimensional space spanned by the top eigenvectors of the covariance matrix. While these basis vectors are optimal in a statistical sense, PCA has been criticized for being less faithful to the nature of the data at hand. For instance, data mining practitioners often tend to assign a “physical” meaning to the resulting factors. Such reification must be based on an intimate knowledge of the application domain and can often not be justified from mathematics. This also holds for other techniques such as Non-Negative Matrix Factorization due to [35] and K-means clustering which implicitly performs matrix factorization. More importantly, classical approaches pose the difficulty of characterizing sophisticated patterns of data point distributions in a unified parametric and interpretable form. This is generally intractable [36].

**Figure 7:**
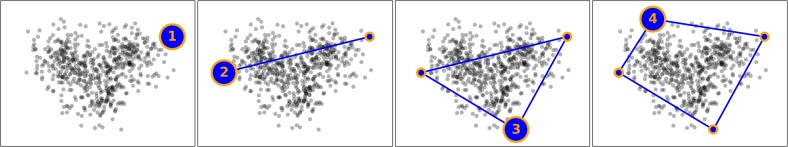
Example in which way Simplex Volume Maximization iteratively determines basis vectors for interpretable matrix factorization.

An alternative are interpretable matrix factorizations methods, which compute low rank approximations from selected columns of a data matrix [37]. They are increasingly popular in the data mining community [33, 38–43] since they preserve properties such as sparseness or non-negativity and were successfully applied in many important applications, e.g. fraud detection, fMRI segmentation, drought detection in plants or collaborative filtering. Here we consider interpretable data driven methods in the context of convexity constrained matrix factorization that can be written as

$$\begin{array}{c} E=\|X-XGH{\|}_{F}^{2}=\|X-WH{\|}_{F}^{2}\\ s.t.g_{i}\text{ binary},\;\sum_{j}g_{ij}=1,\\ h_{i}\underline{≻}0,\;\sum_{j}h_{ij}=1, \end{array}$$ (2)

where ‖⋅‖_*F*_ denote the Forbenius norm, the values in matrix **H** have non-negativity constraints and the matrix **G** is restricted to be binary. Consequently, the basis vectors **W** are real data points, i.e. columns of the data matrix **X** that represent actual observations. Therefore, they have naturally a biological meaning and can easily be interpreted by a domain expert. As pointed out by Cutler and Breiman [44], basis vectors in convexity constrained matrix factorization correspond to the most extreme, rather than to average columns. Moreover, it was shown that a good subset of columns maximize their volume [45,46]. Therefore, we determine *c* columns from the input data matrix **X** as basis vectors **W**, such that the volume of the simplex spanned by the columns of **W**, is maximized. However, the maximum-volume criterion is provably NP-hard [45]. An approximation called Simplex Volume Maximization (SiVM), was introduced by Thurau *et al.* (2012) [33] and empirically proven to be feasible and reliable. The authors presented an efficient and linear time greedy approach that iteratively determines basis vectors using the notion of distance geometry. An example of how SiVM works is illustrated in Fig. 7 on a synthetic data set consisting of three Gaussians components. For the first data points to be selected, we simply take the two points which are most likely furthest away from each other. Pairwise distances computed in one iteration can be reused in later iterations so that, for retrieving *c* columns, we need to compute distances from the last selected column to all other data points exactly *c* + 1 times. As *c* is constant, we have an overall effort of O(n) since the coefficients in **H** can computed in a single pass over the data set solving a constrained quadratic program [47] of fixed dimensions per datapoint. We refer to [33] for more details.

We make use of SiVM in (Step 1) of our approach in order to determine the most extreme columns (hyperspectral signatures) and to use them as basis vectors **W** in Eq. (2). For the plant data, however, before computing the decomposition, we stacked all matrices of hyperspectral images. On the resulting matrix we first selected *c* = 25 extreme signatures per data set (VNIR and SWIR) that form the matrix of basis vectors **W**. This allows us to capture global dependencies. Since the basis vectors correspond to actual data points, they can be identified as signatures of a diseased, dry or healthy leaf. Given the matrix **W**, we then computed the coefficients **H**, i.e. the coordinates of all signatures in the space spanned by the extreme data points, in a single pass over the entire data set. Experiments were run on a standard Intel Quad-Core CPU with 3.06 GHz and 8 GB main memory. For the larger VNIR data, it took about 80 minutes on only a single core to determine the *c* basis vectors; for the smaller SWIR data set, it took less than 1 minute. The computation of the reconstruction matrix **H** for all images took less than 2 hours by using all cores. However, computing the factorization was the most time consuming part of the cascade, whereas performing the remaining steps was a matter of minutes in each case.

#### Steps 2–3: Archetypal Bayes Factors

One of our goals is to find the archetypal hyperspectral signatures for a disease. Up to now, hyperspectral signatures are represented by means of convex combinations **H** of extreme signatures **W** discovered by stacking the hyperspectral signatures of all plants and diseases. Therefore, we next address the question of how to turn these “global” reconstructions into disease-specific reconstructions? That is, how to make use of the available disease label of each hyperspecral image?

**Step 2: From Reconstructions to Distributions on the Simplex**: The resulting reconstructions **H** are proportions that sum to one and describe the relative contribution of each of the *c* columns in **W** to a hyperspectral pixel. From a geometric point of view, the columns **h**_1_, …,**h**_*n*_ of **H** can be considered as data points residing in a simplex spanned by **W** so that there are natural parametric distributions for **h**_*i*_ on the simplex. Probably the best known one is the Dirichlet distribution

$$Dir(h_{i}|\alpha )=\frac{\Gamma (\sum_{j=1}^{c}\alpha_{j})}{\prod_{j=1}^{c}\Gamma (\alpha_{j})}\prod_{j=1}^{c}h_{ij}^{\alpha_{j}-1},$$

which is parametrized by a vector $\alpha =(\alpha_{1},\alpha_{2},\ldots ,\alpha_{c})$ and where Γ denotes the gamma function, see Fig. 2 (Step 2) for an illustration. Dirichlets naturally enforce convexity constraints on the reconstructions, 0 ≤ *h*_*ij*_ ≤ 1 and $\sum_{j=1}^{c}h_{ij}=1$, so that changing one *h*_*ij*_ impacts all other *h*_*ik*_. To estimate the parameters *α* from the reconstructions **H**_*i*_, we follow a maximum-likelihood approach [48]. The simplex distribution induced by the reconstructions **H**_*i*_ per class/disease *i* results without having made any priori assumption as to the underlying distribution next to the distance measure used within SiVM.

**Step 3: Using Labels to Select Disease Archetypes:** Having distributions at hand opens the door to statistical machine learning. In particular, we make use of the label information. Given the Dirichlet distributions with parameters *α*_*k*_ for a disease *k* and *α*_*h*_ for the healthy plant, the task is to decide which is the best distribution describing signatures **S**_*k*_ within a diseased plant *k*. To this end, we apply Bayes factor [49, 50]: $BF(S)=\frac{P(S|{ℋ}_{i})}{P(S|{ℋ}_{j})}\;$. Here, two distributions ${ℋ}_{i}$ and ${ℋ}_{j}$ are compared by forming the posterior odds. Note that we assume that the prior over the models is uniform, i.e. P(ℋ) is a constant. Since our models are distributions of classes learned on the simplex we are dealing with the simplest case where Bayes factors coincide with the likelihood ratio [49]. Specifically, we advocate the use of log-likelihood ratio together with Dirichlets:

$$LLR{\left(S\right)}_{ij}=\text{log}\;Dir(S|\alpha_{i})-\text{log}\;Dir(S|\alpha_{j}).$$ (3)

The interpretation of *LLR*_*ij*_ w.r.t. class *i* is as follows: if *LLR*_*ij*_ ⩽ log(1) there is no difference to *j* but if *LLR*_*ij*_ > log(1) there are a differences, and, the larger the *LLR*_*ij*_, the more pronounced the differences. Fig. 2 (Step 3) illustrates this using three overlapping Gaussians; the darker data points for Gaussian 2 denote a higher difference, i.e. a higher min_*j*_ *LLR*_2*j*_ value for *j* = 1,3. Using the *LLR*, we can select highly differential archetypal signatures for each disease. Examples of diseased spots and their differences to a healthy plant, which were selected in (Step 3) of our approach are shown in Fig. 4. The more yellow or red a pixel in this figure is, the more it differs.

#### Step 4: Dirichlet Aggregation Regression and Mean Archetypal Signatures

Given sequences of disease distributions, we are interested in capturing their dynamics over time. To capture the dynamics of a single disease, we make use of Dirichlet-Aggregation Regression (DAR) [15]. DAR allows for modeling and predicting the distribution over disease archetypal signatures at any time. Specifically, DAR assumes a Bayesian perspective and supposes that the reconstruction $h_{t}^{k}$, computed for a single pixel in hyperspectral image of a plant *k* at time *t*, was generated from a hidden Dirichlet distribution parameterized by the variable $\alpha_{t}^{k}={\left[\alpha_{t1}^{k},\ldots ,\alpha_{tc}^{k}\right]}^{T}$, where *c* denotes the reconstruction dimension. To do so, it puts a Gaussian process [51] prior on the Dirichlet distributions induced on the simplex spanned by the extremes. For more details we refer to [15].

DAR is realized in (Step 4) of our approach (cf. Fig. 2). In order to sketch the hyperspectral dynamics of diseased plants, we first predict Dirichlets $\alpha_{t}^{k}$ using the GP for *T* intermediate time points and compute the Dirichlet mean archetypal signature $\varsigma_{t}^{k}=[\varsigma_{t1}^{k}\ldots ,\varsigma_{t\Lambda }^{k}]$ for a plant *k* at time *t* as

$$\varsigma_{t\lambda }^{k}=\sum_{i=1}^{c}E[\alpha_{ti}^{k}]W_{i\lambda }$$ (4)

where $E[\alpha_{ti}^{k}]=\alpha_{ti}^{k}/(\sum_{j}\alpha_{tj}^{k})$ and $W\in {\mathbb{R}}^{\text{Λ}\times c}$ contains *c* extreme signatures selected across all plants and time steps and *λ* denotes the current dimension (wavelength in hyperspectral signature). Examples of mean archetypal signatures for different diseased plants are shown in Fig. 3. As discussed above, they allow us to automatically sketch the hyperspectral dynamics of plant diseases, as illustrated in Fig. 5 and Fig. 6. Next, we discuss how to draw such interpretable summaries.

#### Step 5: Mapping Hyperspectral Dynamics of Diseased Plants

Given the *K* sequences of automatically assessed mean disease archetypal signatures for different stages of diseases progression (where *K* is the number of diseases), we are left with summarizing their dynamics, highlighting interesting time points, and describing the behavior of disease symptoms. We distinguish between multiple and single sketches, which we formally define as

**Definition 1 (Summary)** *A sketch* S *is a directed connected graph G* = (*V*,*E*), *where V is a set of nodes and E is a set of edges, and* Π *a set of D paths in G. Each edge e* ∈ *E belongs to at least one path* Π *and each node v_i_* ∈ *V consists of at most D incoming and D outgoing edges*.

Here, *D* denotes the number of different paths, say, diseases. A single sketch follows from this definition by setting *D* = 1 and thus consists of a sketch summarizing interesting time points of one particular diseased plant.

**Single Sketches:** To create a single sketch we are looking for a segmentation of ordered objects in *B* equally weighted bins which preserves the original ordering of the objects. Given a matrix $X\in {\mathbb{R}}^{K\times N}$ where the columns denote the hyperspectral signatures representing different stages of diseases progression, we can achieve a segmentation into *B* bins as follows: First, we compute the distances of consecutive spectra (columns) using their Euclidean distance and compute the average bin size as

$$\delta =\frac{1}{B}\sum_{i=1}^{N-1}d_{i(i+1)}(X)\;\text{where}\;d_{ij}(X)=\sqrt{\sum_{k=1}^{K}{\left(x_{i}^{k}-x_{j}^{k}\right)}^{2}}$$ (5)

Then, we fill the *B*−1 bins successively with the objects according to the bin size *δ*. The last bin is filled with the remaining objects. Drawing a single sketch is now simple. Each node denotes the begin (resp. end) of a period, and the length of the edge *e*_*b*_ between two consecutive nodes *v*_*b*_ and *v*_*b*+1_ is set relatively to the length of the period covered by the objects in bin *b*. Thus, the shorter a part, the higher the impact of the corresponding period. This is illustrated in Fig. 5 for several diseased plants. Each sketch highlights the interesting periods of a disease, where a small edge denotes a period of high impact (change in hyperspectral signature).

The limitation of a single sketch, however, is that it only represents the progression of a single diseased plant. In order to uncover (dis-)similarities in progression and behavior of several diseased plants, we thus consider multiple sketches.

**Multiple Sketches via Metro Maps of Diseased Plants:** As an addition to the above sketches, we provide an abstract map showing spectral dynamics and changes during the pathogenesis. Here, we first embed hyperspectral images of plants, represented by means archetypal signatures, in a low dimensional Euclidean space. classical approaches for doing so are multidimensional scaling (MDS) [52] and IsoMap [53]. IsoMap first creates a graph by connecting each object to *l* of its neighbors and then considers path lengths in the graph for embedding using multidimensional scaling. Nevertheless, when analyzing such embeddings, it is easy to focus on a narrow aspect such as how close two plants are at a specific point in time and to lose track of the big picture. As small difference in distances are not of great importance overall, we abstract the embedding further into a multiple sketch, which is motivated by the idea of metro maps, the schematic diagrams of public transport networks. To draw a metro map of diseased plants, we used the approach described by Nöllenburg and Wolff [26] which takes as input the two dimensional embedding computed by, e.g. by MDS or IsoMap, and turns into into a metro map by solving a linear program. We refer to [26] for more details.

A modified version of IsoMap that makes use of temporal information was already used for the creation of simplex traces [14]. Unlike in classical MDS (which is employed by IsoMap), we here seek a solution for the minimization problem using any gradient-based optimization method, e.g. via quasi Newton methods [54] or via the SMACOF algorithm for Stress functions [52] which is based on iterative majorization. In particular, we seek a configuration of points representing the plants such that the distances between these points matches their similarity as close as possible

$$\sigma_{r}(X)=\sum_{i,j}a_{ij}{\left(d_{ij}(X)-\delta_{ij}\right)}^{2},$$ (6)

where *δ*_*ij*_ denotes the dissimilarity between plant *i* and *j*, **X** denotes a point configuration, the weights in *a*_*ij*_ indicate if the value *δ*_*ij*_ is missing (*a*_*ij*_ = 0, imposing no restrictions on the configuration in **X**) or are known (*a*_*ij*_ = 1), and *d*_*ij*_(**X**) is the Euclidean distance used in Eq. (5). Thus, matrix **A** can indicate whether two matrices belong to the same time slice or plant/sequence but at consecutive days. Additionally, we may fix some of the points to predefined coordinates. That is —(i) since all plants were healthy on the first day, we fix the coordinates of the first measurements, and (ii) since we have observations over time, the *x*-coordinates are set to 1,2,3, …,*t*— we find an embedding by adjusting only the unknown points $\hat{x}\in X$ such that the Stress function is minimized.

**Figure 8:**
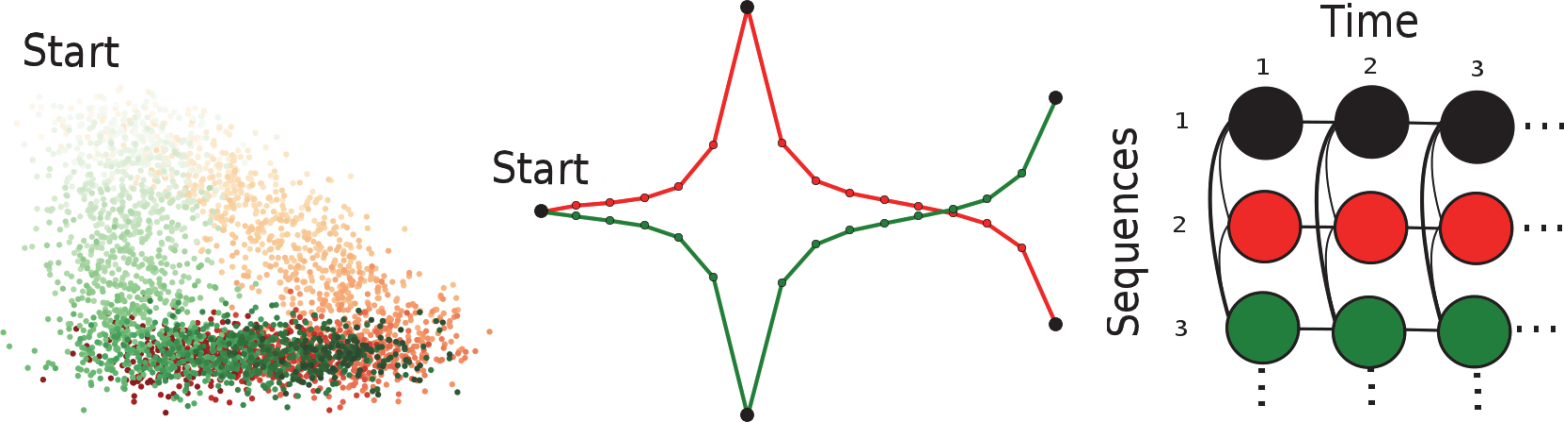
Example for multiple sketches on a synthetic data set. The data set was generated by two moving Gaussians (green and red) where the brightness encodes time (the darker, the later) (left). The corresponding embedding using MDS (middle), the black dots were fixed on both dimensions whereas for the green and red dots only the x-axis coordinates were fixed (*x*_*i*_ = *t*_*i*_, where t is the temporal information) and y-coordinates were unknown (learned by MDS). (right) The connection graph defining the weight matrix *W* between different states of N sequences over time, e.g. the moving Gaussians: *w*_*ij*_ = 1 if the two elements *i* and *j* belong to the same time slice (column) or to the same entity (raw or the same color), say moving Gaussian, but at consecutive time points.

To see that this two-step approach is able to sketch dynamics in general, consider the two moving Gaussians shown in Fig. 8 (left). Both Gaussians start in the upper left corner, then, the green one moves in L-shape to the bottom right corner, and the red one moves first to the bottom right corner and then to the bottom left corner. Each Gaussian was evaluated at 16 positions sampling 100 data points. To obtain an embedding, we took the mean coordinates of each Gaussian. The matrix **A** was defined as described above by preserving the temporal information (see e.g. Fig. 8 right). The resulting embedding is shown in Fig. 8 (middle), where the black dots were fixed on both dimensions whereas for the remaining green and red dots only the x-axis coordinates were fixed (*x*_*i*_ = *t*_*i*_, where t is the temporal information) and y-coordinates were unknown (learned by MDS). This way, the behavior of both Gaussians is captured while respecting their similarities. To compute a sketch of this embedding, we first connect two elements *i* and *j* if *d*_*ij*_(**X**) < *ε*, for a small threshold *ε* > 0. The resulting metro map is illustrated in Fig. 2 (Step 5) and provides an abstract summary describing the behavior of two moving Gaussians.

## Conclusions

In this paper, we present the first fast and reliable data-driven method for sketching complex hyperspectral dynamics in plant science and for plant phenotyping. Disease archetypal signatures as well as the sketches of hyperspectral dynamics conform to plant physiological knowledge, providing interpretable summaries of disease progression via single sketches and metro maps of diseased plants.

Our work provides several interesting avenues for future work. Clearly, other plant pathogen systems and different genotypes under abiotic or biotic stress should be investigated. Another interesting direction is to develop part based sketches, for instance, by applying regularized latent Dirichlet allocation [13]. Additionally the benefits of using disease archetypal signatures in early detection or classification tasks may be further investigated.

## Supporting Information

S1 File Basis vectors determined by Simplex Volume Maximization. The basis vectors correspond to the most extreme signatures for the VNIR and SWIR ranges. Each row corresponds to a wavelength and column to a basis vector. The entries contain the reflectance information at different spectral bands.
(ZIP)

S2 File Dirichlet parameter predicted by Gaussian process. Each file contains the information for a specific disease or control plants for the VNIR and SWIR ranges. The Dirichlet parameter were used to compute mean archetypal signatures which are the basis for creating the metro maps of diseased plants.
(ZIP)

S1 Fig Single sketches of diseased plants over time for VNIR range. (TIFF)

S2 Fig Single sketches of diseased plants over time for SWIR range. (TIFF)
