# Supplementary figures and images for: Metro Maps of Plant Disease Dynamics—Automated Mining of Differences Using Hyperspectral Images
Source: PLoS One. 2015 Jan 26;10(1):e0116902. doi: 10.1371/journal.pone.0116902 (PMC4306502; doi:10.1371/journal.pone.0116902)

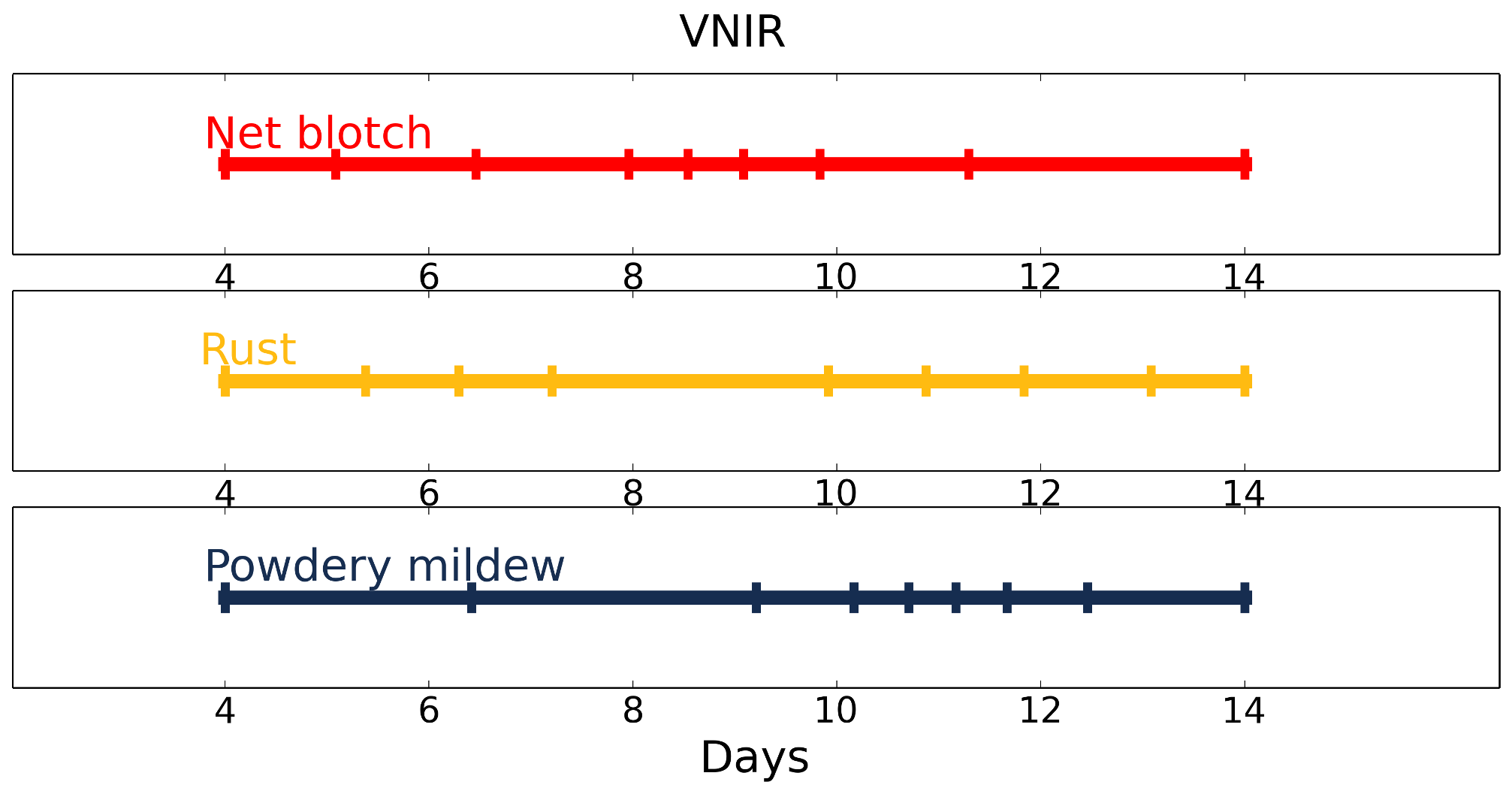

Supplement: S1 Fig — (TIFF) [file pone.0116902.s003.tiff]

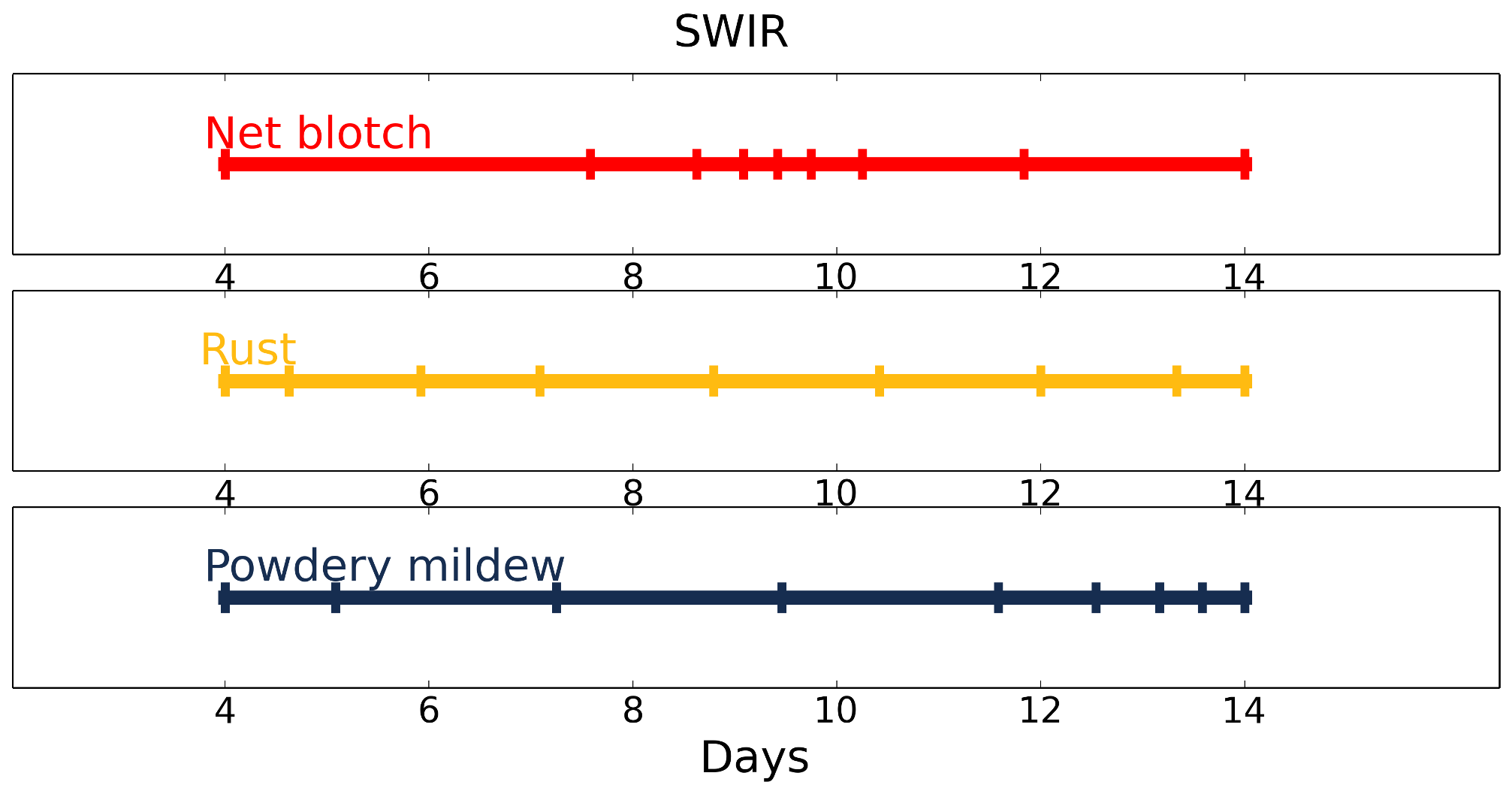

Supplement: S2 Fig — (TIFF) [file pone.0116902.s004.tiff]
